# Evaluation of the protective effect of *Curcuma longa* and PPARγ agonist, pioglitazone on paraquat‐induced lung injury in rats

**DOI:** 10.1002/iid3.70001

**Published:** 2024-08-22

**Authors:** Mohammad Hossein Eshaghi Ghalibaf, Mohammad Ehsan Taghavi zadeh Yazdi, Mona Mansourian, Nema Mohammadian Roshan, Mohammad Hossein Boskabady

**Affiliations:** ^1^ Applied Biomedical Research Center Mashhad University of Medical Sciences Mashhad Iran; ^2^ Department of Physiology, School of Medicine Mashhad University of Medical Sciences Mashhad Iran; ^3^ Student Research Committee, Faculty of Medicine Mashhad University of Medical Sciences Mashhad Iran; ^4^ Department of Pathology, Faculty of Medicine Mashhad University of Medical Sciences Mashhad Iran

**Keywords:** *Curcuma longa*, inflammation, lung injury, oxidative stress, paraquat

## Abstract

**Background:**

The inhalation of paraquat (PQ), one of the most widely used herbicides in the world, can result in lung injury. *Curcuma longa* (Cl) has long history in traditional and folk medicine for the treatment of a wide range of disorders including respiratory diseases.

**Aim:**

The aim of the present work was to evaluate the preventive effect of Cl on inhaled PQ‐induced lung injury in rats.

**Methods:**

Male Wistar rats were divided into 8 groups (*n* = 7), one group exposed to saline (control) and other groups exposed to PQ aerosol. Saline (PQ), Cl extract, (two doses), curcumin (Cu), pioglitazone (Pio), and the combination of Cl‐L + Pio and dexamethasone (Dex) were administered during the exposure period to PQ. Total and differential white blood cell (WBC) counts, oxidant and antioxidant indicators in the bronchoalveolar lavage (BALF), interleukin (IL)‐10, and tumor necrosis alpha (TNF‐α) levels in the lung tissues, lung histologic lesions score, and air way responsiveness to methacholine were evaluated.

**Results:**

WBC counts (Total and differential), malondialdehyde level, tracheal responsiveness (TR), IL‐10, TNF‐α and histopathological changes of the lung were markedly elevated but total thiol content and the activities of catalase and superoxide dismutase were decreased in the BALF in the PQ group. Both doses of Cl, Cu, Pio, Cl‐L + Pio, and Dex markedly improved all measured variables in comparison with the PQ group.

**Conclusion:**

CI, Pio, and Cl‐L + Pio improved PQ‐induced lung inflammation and oxidative damage comparable with the effects of Dex.

## Graphical abstract

The effect of *Curcuma longa* (Cl) on inhaled paraquat (PQ)‐induced lung injury was examined. CI and pioglitazone ameliorated lung injury induced by inhaled PQ. CI improved lung inflammation similar to dexamethasone. Synergic effect of pioglitazone and CI was shown. Peroxisome proliferator‐activated receptors mediated effects of CI was suggested.

## 1 INTRODUCTION

Paraquat (PQ) is one of the most generally employed herbicides in the world ^1^ due to its low price and ease of use.^2^, ^3^ Acute PQ poisoning causes lung injury and lung fibrosis ^4^, ^5^ and the most public ways of exposure to PQ are through inhalation and dermal.^6^, ^7^ Administration of PQ can increase inflammatory factors such as tumor necrosis alpha (TNF‐α).^8^ Oxidative stress, due to the cytotoxic property of PQ, causes the occurrence of immune response and can trigger inflammation as a pathological condition. Based on this, most commonly drugs that are used in clinical management of PQ poisoning, such as methylprednisolone and cyclophosphamide, are prescribed with the aim of modulating the inflammatory response in PQ poisoned patients.^9^, ^10^ The pharmacology industries are hence in need of reliable and applied biomedical methods to neutralize the toxic effects of this herbicide. Phyto‐medicine has been used for remedial purposes throughout history.^11^, ^12^ Nowadays, more attention is given to the investigation of plants and their derivatives for the treatment of diverse diseases.^13^ Phyto‐medicine signifies a collection of remedial information that is intensely rooted in a civilization and designed as a source of the primary form of pharmacopeias, which was based in a great part on natural products.^14^ Turmeric with the scientific name, *Curcuma longa* (Cl) is one of the most generally studied plants.^15^ Turmeric known as Zard‐choose in Persian, has been used in many countries since ancient times ^16^, ^17^ and it improves the flavor and color of foods.^18^ Many people across the world use turmeric and its different components as a traditional medicine to remedy human diseases with special reference to Iran, China, and India. Turmeric has been extensively applied for a wide range of disorders including diabetes, asthma chronic obstructive pulmonary diseases, cardiovascular, and inflammation.^19^, ^20^ Moreover, turmeric oil contains essential fatty acids and has antifungal, anti‐mutagenic, and antibacterial properties. Curcumin (Cu) as a polyphenolic constituent from the rhizome of the Cl has been introduced as a potent anti‐inflammatory and antioxidant compound.^21^ Recent reports have demonstrated that Cu can mitigate lung damage in various experimental models such as viral infection induced respiratory failure,^22^ mechanical ventilation‐induced lung injury in rats,^23^ and animal model of asthma.^24^ These traditional uses of Cl highlight its significance in promoting respiratory health and supporting overall well‐being.

A part of the nuclear hormone receptor family is made up of PPARs (Peroxisome proliferator‐activated receptors).^25^ PPAR got its name because it responds to peroxisome proliferators and comes in three different isoforms: alpha, beta, and gamma.^26^ PPAR‐γ is a known member of the nuclear receptor superfamily that regulates the expression of genes related to the metabolism of lipids and glucose.^27^ The involvement of PPAR‐γ in immune response regulation and anti‐inflammatory functions is achieved by regulating macrophage activation and suppressing proinflammatory genes, leading to the suppression of cytokine expression and inhibition of the nuclear factor kappa B (NF‐κB) pathway, highlighting its importance.^28^, ^29^, ^30^ There are evidence regarding the effects of CL and Cu on respiratory and allergic disorders and their anti‐inflammatory, antioxidant and immunodulatory effects.^31^ In addition, the treatment effects of CL on PQ‐induced lung and systemic injuries were shown by exposing animal to PQ aerosols for 16 days and treated them with CL after PQ exposure for another 16 days.^15^ However, the preventive effect of CL on PQ‐induced lung injury was not shown. Therefore, in this study, the preventive effects of turmeric against PQ aerosol–induced lung injury in rats are assessed. Rats were exposed to inhaled PQ for 8 times in 16 days and treated with turmeric extract during the exposing period to examine their preventive effect. Total and differential white blood cell (WBC) counts as a marker of inflammation, and oxidant‐antioxidant markers, in the bronchoalveolar lavage (BALF), the level of lung tissue cytokines, lung pathological changes, and airway responsiveness to methacholine were measured.

## 2 MATERIAL AND METHODS

### 2.1 Chemical and reagent

CI longa were purchased from a local herbal drugstore in Mashhad, Razavi Khorasan Province, Iran and identified by botanists in the herbarium center of Ferdowsi University of Mashhad. The extract of commercially obtained Cl was prepared as described previously 31. IL‐10 and TNF‐alpha assay kit was purchased from Karmania. Cu was purchased from Sami labs LTD. Pioglitazone was obtained from a Pharmaceutical Company (Samisaz) in (Mashhad, Razavi Khorasan Province). Also, PQ was prepared from Sigma‐Aldrich Chemical Co. Ethanol 96% was purchased from betagene laboratory equipment. Dexamethasone was obtained from Sigma Aldrich Chemical Co. (MOUSA).

### 2.2 Inclusion/exclusion criteria

Inclusion criteria: Wistar rats (age: 50–90 days, weight: 200−250 g) were used for this study. Exclusion criteria: Pups with weight less than 180 g and higher than 250 g, animals with unexplained weight loss, rats with symptoms of acute respiratory syndrome or infection.

### 2.3 Experimental procedure

Fifty‐six Wistar rats (males weighing 200−250 g) were obtained from the animal house of Mashhad University of Medical Sciences (MUMS) and placed in clean ventilated cages The animals were maintained at 25 ± 2°C temperature, 52 ± 2% relative humidity, and 12 h of light/dark cycle with free access to food and water.

Animals were randomly distributed in 8 groups (*n* = 7 in each group) including:

(1) Control group: Rats were exposed to saline aerosol 8 times in every other day order, each day for 30 min.

(2) PQ group: Rats were exposed to PQ aerosol, 8 times in every other day order, each day for 30 min.^32^, ^33^

(3, 4) Cl extract groups: Rats were exposed to PQ aerosol and received Cl extract (150 or 600 mg/kg) daily by gavage during 16 days.^32^, ^33^

**Figure 1:**
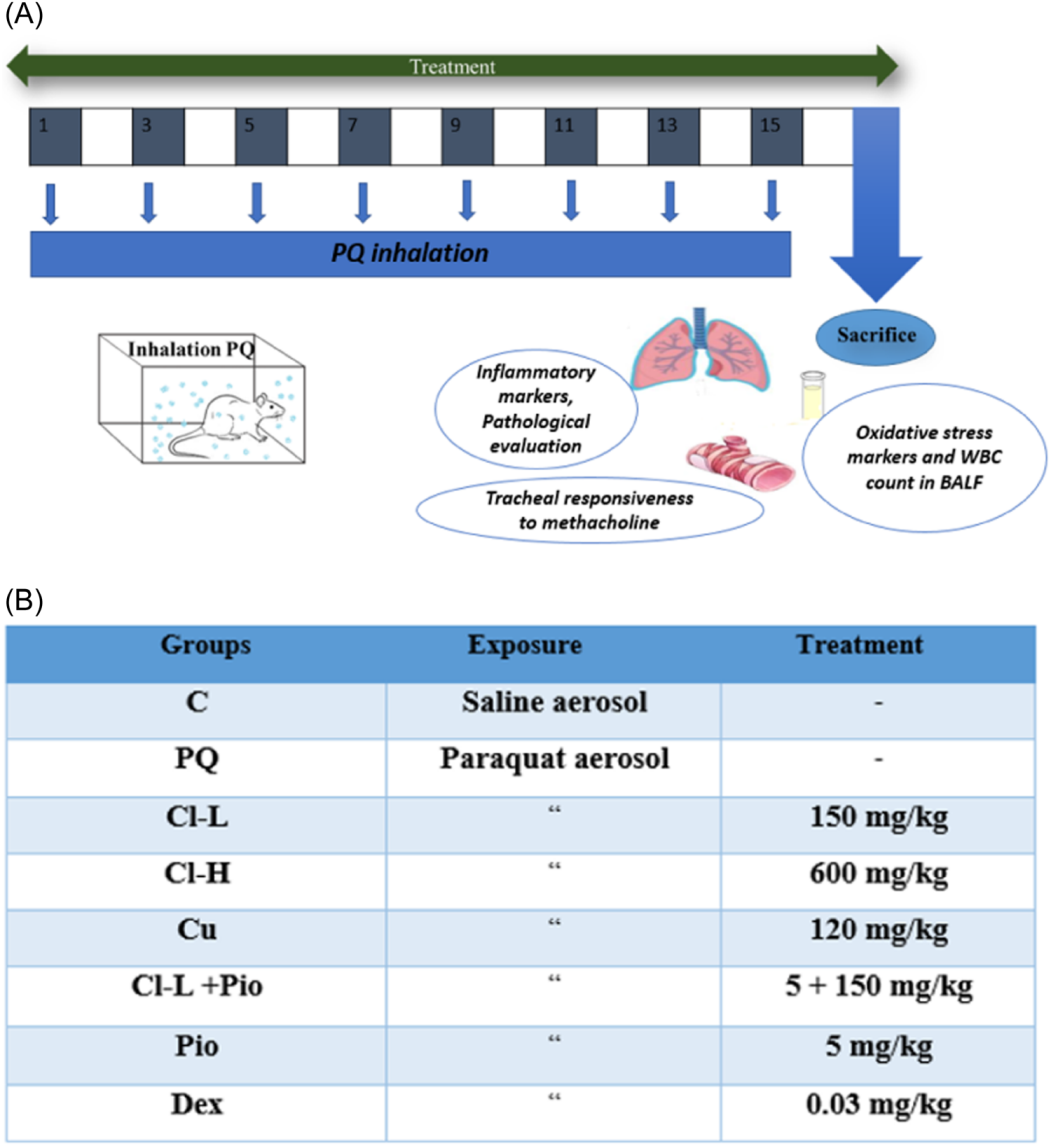
Protocol of animal exposure to PQ aerosol (A) and treated groups (B). In the control group (group C), rats were exposed to saline aerosol. Seven groups were exposed to PQ aerosol at the dose of 54 mg/m^3^ and treated with: (1) Salin (PQ group), (2 and 3) 150 and 600 mg/kg/day of Cl extract (Cl‐L and Cl‐H groups), (4) 120 mg/kg of curcumin (Cu), (5) 5 mg/kg/day of pioglitazone (Pio), (6) Combination of Pio + Cl‐L (Pio + Cl‐L), and (7) 0.03 mg/kg/day of dexamethasone (Dex) for 16 days during exposure to PQ by gavage except for Pio which was administered by i.p. Rats were exposed to saline or PQ aerosol, 8 times in every other day order, each day for 30 min.

(5) Cu group: Rats were exposed to PQ aerosol and received Cu (120 mg/kg) daily by gavage during 16 days.^32^, ^33^

(6) Cl –L + Pio group: Rats were exposed to PQ aerosol and received Cl‐l (150 mg/kg) and Pio (5 mg/kg) combination daily by gavage during 16 days.

(7) Pio group: Rats were exposed to PQ aerosol and received Pio (5 mg/kg) daily by gavage during 16 days.^32^, ^33^

(8) Dex group: Rats were exposed to PQ aerosol and received Dex (0.03 mg/kg) daily by gavage during 16 days. Figure 1 shows the protocol of animal exposure to PQ aerosol and a table of the study groups.^32^, ^33^

On Day 17 (at the end of the study period), after deep anesthesia induction by ketamine (50 mg/kg) and xylazine (5 mg/kg) combination, the rats were killed. The study was approved by the ethics committee of MUMS (981,734). In addition, animal experiments were done by the United Kingdom. Animals Act, 1986, EU Directive 2010/63/EU for animal experiments. Animal testing experiments also complied with the ARRIVE guidelines.

### 2.4 Biochemical measurement

Five millliter of BALF was prepared from the right lung. Cellular analysis of the BALF was performed based on previous reports.^34^ BALF was centrifuged at 2540 rpm for 10 min, and stored at −70°C until measurement of oxidative stress markers. Previous protocols were followed to determine the content of malondialdehyde (MDA), activities of superoxide dismutase (SOD) and catalase (CAT), and thiol group levels.^35^

Briefly, colorimetric assay was used for measuring MDA level in BALF. BALF supernatant was incubated with reaction mixture containing thiobarbituric acid and tricarboxylic acid and centrifuged at 12,000 rpm for 5 min. Absorbance of the supernatant was read at 532 nm and was reported as μM. For Thiol assay, the combination of Na2EDTA, 5,5'‐Dithiobis(2‐nitrobenzoic acid (DTNB), and BALF supernatant was incubated and the absorbance of product was recorded at 412 nm and expressed as μM. Also, the activity of SOD was recorded at 560 nm which is based on its ability to inhibit the photochemical reduction of NBT by O_2_^•−^. In addition, activity of CAT was analyzed by determining its ability to convert H2O2 into water and oxygen. In this method, the rate of decrease of absorbance of BALF supernatant and H_2_O_2_‐phosphate buffer mixture monitored at 240 nm.

Using specific enzyme‐linked immunosorbent assay kits and respecting manufacturing technique recommendation (Karmania pars), the levels of interleukin (IL)‐10, TNF‐α were examined in the lung tissue as previously described method.^32^

### 2.5 Lung histopathological examination

The left lung was removed after animal scarification, and pathological evaluation including interstitial inflammation, emphysema, and lymphocytic infiltration was performed under a light microscope according to a previous study.^34^ Briefly, lung tissue fixation performed through 10% formalin for 72 h. Then, tissue dehydrated in alcohol, cleared in xylene, embedded in paraffin and sectioned into thick Section (5 μm) using a microtome. Finally, lung tissue slices were stained with Hematoxylin and eosin after deparaffinization and studied under light microscopy. Then interstitial inflammation, emphysema, and lymphocyte infiltration were scored from 0 to 4 as previously described.

### 2.6 Air way responsiveness to methacholine

After making an incision in the anterior aspect of the neck area, the trachea ring was removed and organized. Tracheal smooth muscle (TSM) responsiveness to contractile agent (methacholine hydrochloride) was assessed according to the earlier description.^32^

### 2.7 Statistical analysis

Data were analyzed by both one‐way analysis of variance (ANOVA) and the multiple comparison test (Tukey), and results were shown as mean ± standard error of the mean. We consider Values of *p* < .05 as a criteria of statistical significance.

## 3 RESULTS

### 3.1 The results of cellular analysis of BALF

**Figure 2:**
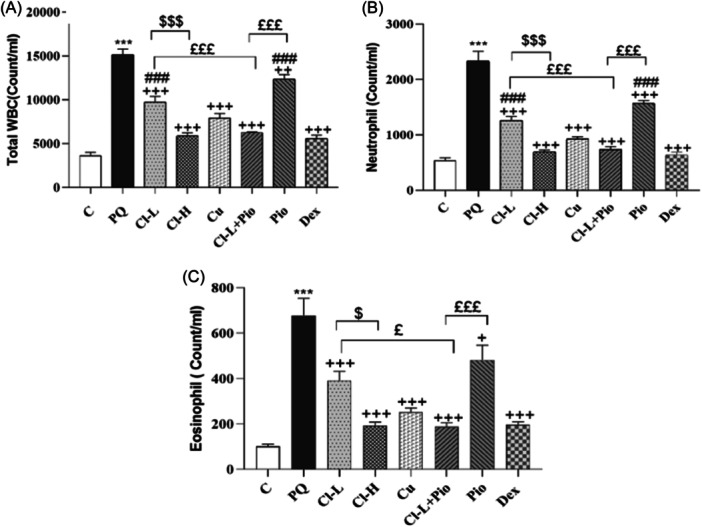
Exposure to PQ affects total WBC (A), neutrophil (B), and eosinophil (C) counts in the BALF (Count/ml) in comparison with the control group (Ctrl) but treatment with 150 and 600 mg/kg/day Cl, (Cl‐L and Cl‐H),120 mg/kg curcumin (Cu), 5 mg/kg pioglitazone (Pio), combination of Cl‐L + Pio or 0.03 mg/kg dexamethasone (Dex) improved them. PQ versus control group; ***p < .001. Treatment groups versus PQ group; + *p* < .05, ++*p* < .01 and +++*p* < .001. Dex versus other treated groups; ≠*p* < .05, ≠≠*p* < .01, and ≠≠≠*p* < .001. Cl‐H versus Cl‐L; $*p* < .05 and $$*p* < .01. Cl‐L + Pio versus Cl‐L and Pio alone; £*p* < .05 and £££*p* < .001. In each group, seveen rats were examined and the results were expressed as mean ± SEM One‐way ANOVA followed by Tukey's multiple comparison test was applied for comparisons among different groups. ANOVA, analysis of variance; PQ, paraquat; SEM, standard error of the mean; WBC, white blood cell.

**Figure 3:**
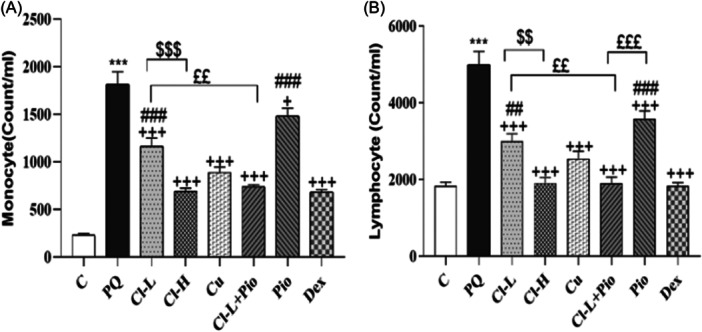
Exposure to PQ affects Monocyte (A)and lymphocyte (B) counts in the BALF (Count/ml) in comparison with the control group (Ctrl) but treatment with 150 and 600 mg/kg/day Cl, (Cl‐L and Cl‐H), 120 mg/kg curcumin (Cu), 5 mg/kg pioglitazone (Pio), combination of Cl‐L + Pio or 0.03 mg/kg dexamethasone (Dex) improved them. PQ versus control group; ***p < .001. Treatment groups versus PQ group; +++*p* < .001. Dex versus other treated groups; ##*p* < .01 and ###*p* < .001. Cl‐H versus Cl‐L; $$*p* < .01 and $$$*p* < .001. Cl‐L + Pio versus Cl‐L and Pio alone; £*p* < .05, ££*p* < .01 and £££*p* < .001. In each group, seven rats were examined and the results were expressed as mean ± SEM One‐way ANOVA followed by Tukey's multiple comparison tests was applied for comparisons among different groups. ANOVA, analysis of variance; PQ, paraquat; SEM, standard error of the mean; WBC, white blood cell.

The number of total and all differential WBC in the BALF of the PQ poisoned group was markedly higher in comparison with the control group (*p* < .001 for all cases). Total WBC, neutrophil, eosinophil, lymphocyte and monocyte counts in all treated experiments were markedly lower than the PQ poisoned group (*p* < .05 to *p* < .001). Total WBC, neutrophil and eosinophil counts recovery in the Cl‐H group was markedly higher than Cl‐L (*p* < .001 for total WBC, neutrophil, and monocyte, *p* < .01 for lymphocyte and *p* < .05 for eosinophil). Recovery of total WBC, neutrophil, eosinophil, monocyte and lymphocyte counts in the Cl‐L + Pio group was markedly higher than in Cl‐L groups (*p* < .001 for total WBC and neutrophil, *p* < .01 for monocyte counts and *p* < .05 for lymphocyte and eosinophil). The therapeutic effects of Cl‐L+ Pio on total WBC, neutrophil, eosinophil, and lymphocyte counts were higher than Pio alone (*p* < .001 for all cases). The effects of Dex on improving total WBC, neutrophil, lymphocyte, and monocyte counts were markedly higher than the both Cl‐L and Pio (*p* < .01 to *p* < .001), (Figures 2 and 3).

### 3.2 The results of oxidative stress indexes

**Figure 4:**
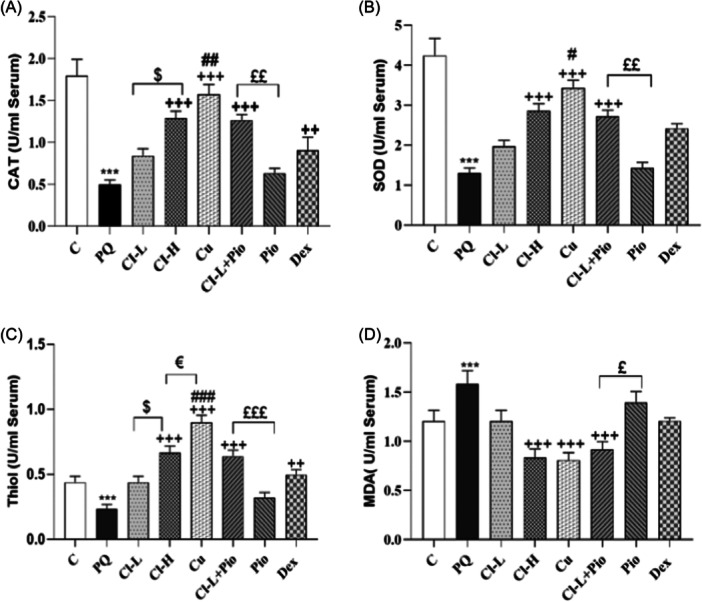
Exposure to PQ affects oxidant and antioxidant biomarkers. CAT (A), SOD (B), Thiol (C), and MDA (D) in the BALF (per mL) in comparison with the control group (Ctrl) but treatment with 150 and 600 mg/kg/day Cl, (Cl‐L and Cl‐H), 120 mg/kg curcumin (Cu), 5 mg/kg pioglitazone (Pio), combination of Cl‐L + Pio or 0.03 mg/kg dexamethasone (Dex) improved them. PQ versus control group; ***p < .001. Treatment groups versus PQ group; +++*p* < .001. Dex versus other treated groups; ##*p* < .01 and ###*p* < .001. Cl‐H versus Cl‐L; $*p* < .05. Cl‐L + Pio versus Cl‐L and Pio alone; £*p* < .05, ££*p* < .01 £££*p* < .001. Cu versus Cl‐H; €*p* < .05. In each group, 7 rats were examined and the results were expressed as mean ± SEM One‐way ANOVA followed by Tukey's multiple comparison tests was applied for comparisons among different groups. ANOVA, analysis of variance; BALF, bronchoalveolar lavage; CAT, catalase; MDA, malondialdehyde; PQ, paraquat; SOD, superoxide dismutase; SEM, standard error of the mean; WBC, white blood cell.

In the PQ poisoned group, the lipid oxidation marker (MDA) level in the BALF was markedly higher than the control group but the level or activity of anti‐oxidant defense agents, total thiol as well as CAT and SOD were markedly lower than control group (*p* < .001 for all cases). Cl‐H, Cu, Cl‐L + Pio, and Dex administration markedly increased CAT activity and level of total thiol in comparison with the PQ poisoned group (*p* < .01 to *p* < .001). SOD activity and MDA level in Cl‐H, Cu, Cl‐L + Pio groups were markedly improved in comparison to the PQ poisoned group (*p* < .001 for all cases). Recovery of CAT activity and total thiol level in the Cl‐H group was markedly higher than Cl‐L (*p* < .05 for both cases). The improvement in CAT and SOD activities, as well as total thiol and MDA levels in the Cl‐L + Pio group, was markedly higher than the Pio group (*p* < .05 to *p* < .001). The improvement in the CAT and SOD activities as well as the total thiol level in the Cu group was markedly higher than the Dex group (*p* < .01 to *p* < .001). Total thiol level in the Cu group was markedly higher than the Cl‐H group (*p* < .05), (Figure 4).

### 3.3 The results of airway sensitivity and responsiveness to methacholine

**Figure 5:**
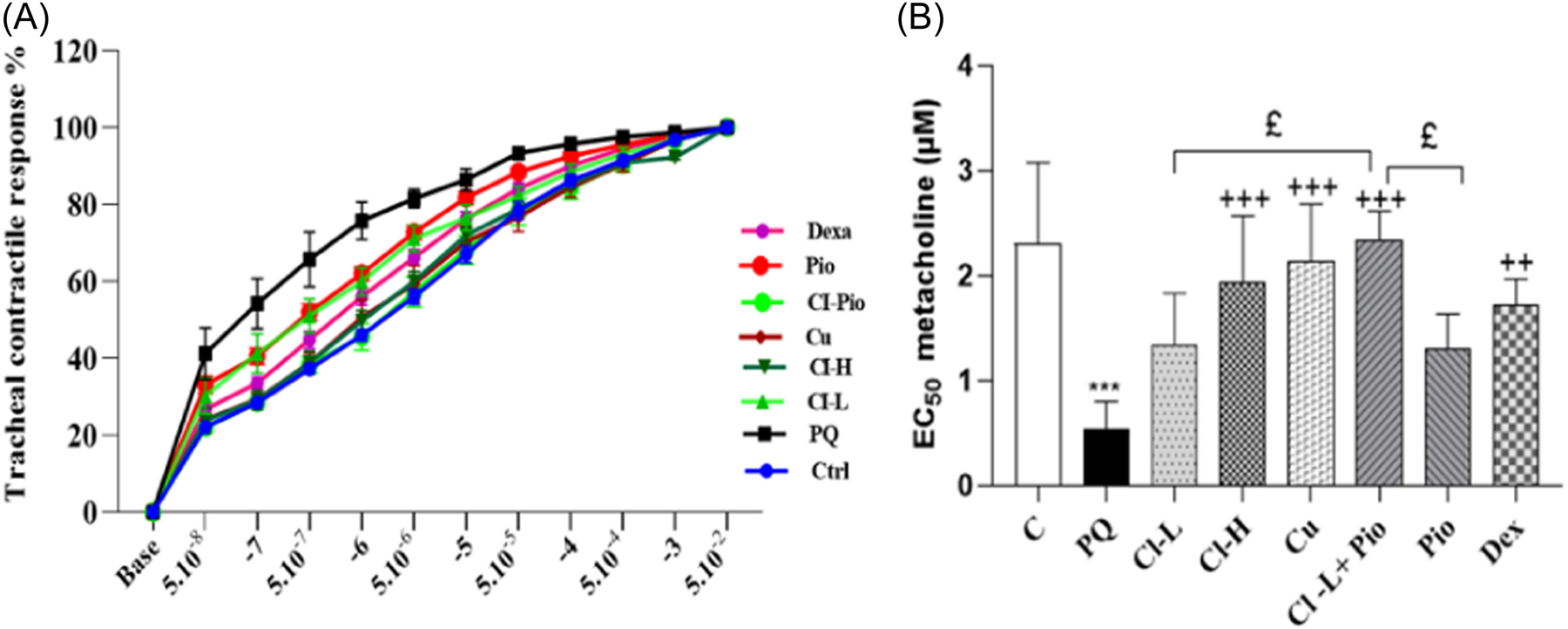
Exposure to PQ affects cumulative concentration‐response curves to methacholine (A) and values of EC50 (B) in comparison with the control group (Ctrl) but treatment with 150 and 600 mg/kg/day Cl, (Cl‐L and Cl‐H), 120 mg/kg curcumin (Cu), 5 mg/kg pioglitazone (Pio), combination of Cl‐L + Pio or 0.03 mg/kg dexamethasone (Dex) improved them. PQ versus control group; ***p < .001. Treatment groups versus PQ group ++*p* < .01, +++*p* < .001. Cl‐L + Pio versus Cl‐L and Pio alone; £*p* < .05. In each group seven rats were examined and the results were expressed as mean ± SEM One‐way ANOVA followed by Tukey's multiple comparisontestst was applied for comparisons among different groups. ANOVA, analysis of variance; PQ, paraquat; SEM, standard error of the mean.

The cumulative concentration‐response curve for methacholine in the PQ poisoned group exhibited a leftward shift, resulting in a notably lower EC50 value for methacholine when compared to the control group (*p* < .001). In all treatment experiments, a shift of a mentioned curve to the right was observed and the values of EC_50_ were markedly increased (*p* < .01 for Dex and *p* < .001 for other groups). The improvement in the EC_50_ in the Cl‐L + Pio group was markedly higher than in the Cl‐L and Pio groups (*p* < .05 in both cases), (Figure 5).

### 3.4 The results of cytokines measurements

**Figure 6:**
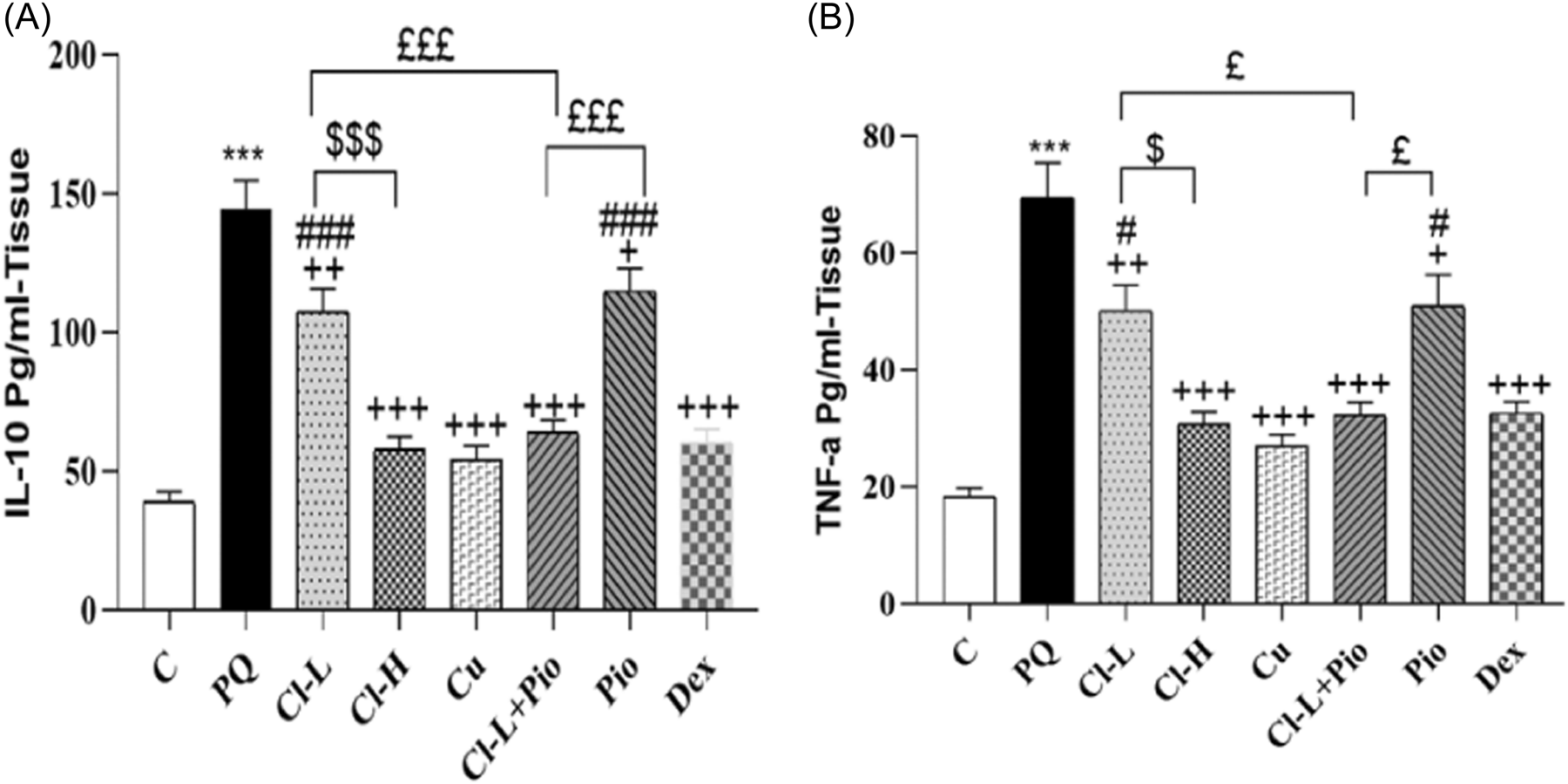
Exposure to PQ affects the levels of IL‐10 (A) and TNF‐a (B) in the lung tissue (per ml) in comparison with the control group (Ctrl) but treatment with 150 and 600 mg/kg/day Cl, (Cl‐L and Cl‐H), 120 mg/kg curcumin (Cu), 5 mg/kg pioglitazone (Pio), a combination of Cl‐L + Pio or 0.03 mg/kg dexamethasone (Dex) improved them. PQ versus control group; ***p < .001. Treatment groups versus PQ group; +++*p* < .001. Dex versus other treated groups; #*p* < .05 and ###*p* < .001. Cl‐H versus Cl‐L; $*p* < .05 and $$$*p* < .001. Cl‐L + Pio versus Cl‐L and Pio alone; £*p* < .05 and £££*p* < .001. In each group, 7 rats were examined and the results were expressed as mean ± SEM One‐way ANOVA followed by Tukey's multiple comparison test was applied for comparisons among different groups. ANOVA, analysis of variance; PQ, paraquat; SEM, standard error of the mean.

IL‐10 and TNF‐a levels in lung tissue of the PQ poisoned group were markedly increased in comparison with the control group (*p* < .001 for both markers). In Cl‐H, Cu, Cl‐L + Pio, and Dex groups, IL‐10 and TNF‐levels were markedly lower than the PQ poisoned group (*p* < .05 to *p* < .001). The therapeutic effect of Cl‐H on IL‐10 and TNF‐α levels was markedly higher in comparison with the Cl‐L group (*p* < .001 and *p* < .05, respectively). IL‐10 levels in the Pio and Cl‐L groups were markedly higher in comparison with the Dex group. The therapeutic effect of Cl‐L + Pio on IL‐10 and TNF‐α levels was markedly higher in comparison with Pio and Cl‐L groups (*p* < .001 for IL‐10 and *p* < .05 for TNF‐α in both cases). (Figure 6).

### 3.5 The results of histopathological evaluation

**Figure 7:**
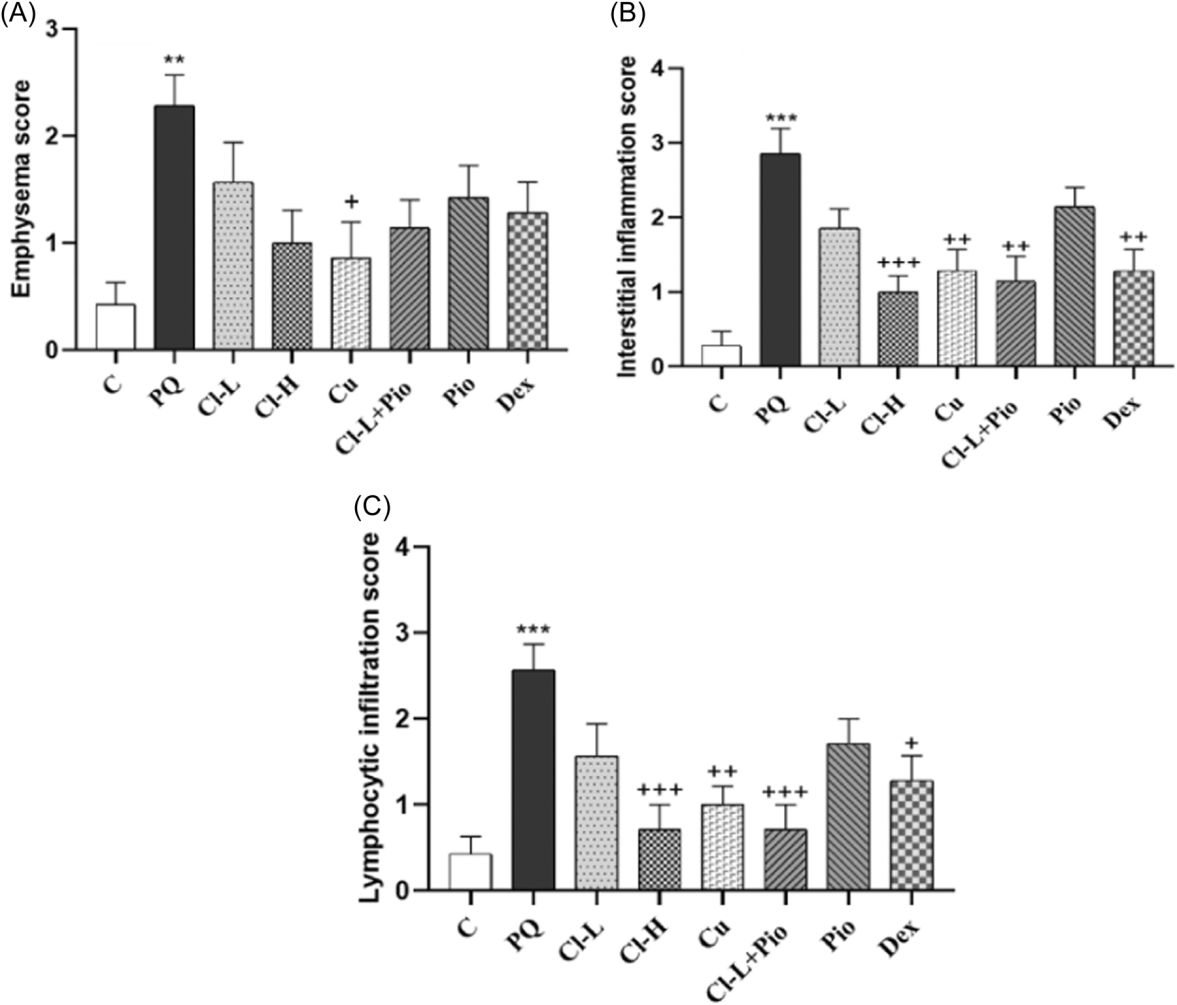
Exposure to PQ affects the score of emphysema (A), interstitial inflammation (B) and lymphocytic infiltration (C) in the lung tissue in comparison with the control group (Ctrl) but treatment with 600 mg/kg/day Cl, (Cl‐H), 120 mg/kg curcumin (Cu), combination of Cl‐L + Pio or 0.03 mg/kg dexamethasone (Dex) improved them. PQ versus control group; ***p < .001 and **p < .01. Treatment groups versus PQ group; +++*p* < .001, ++*p* < .01 and + *p* < .05. In each group, seven rats were examined and the results were expressed as mean ± SEM One‐way ANOVA followed by Tukey's multiple comparison test was applied for comparisons among different groups. ANOVA, analysis of variance; PQ, paraquat; SEM, standard error of the mean.

**Figure 8:**
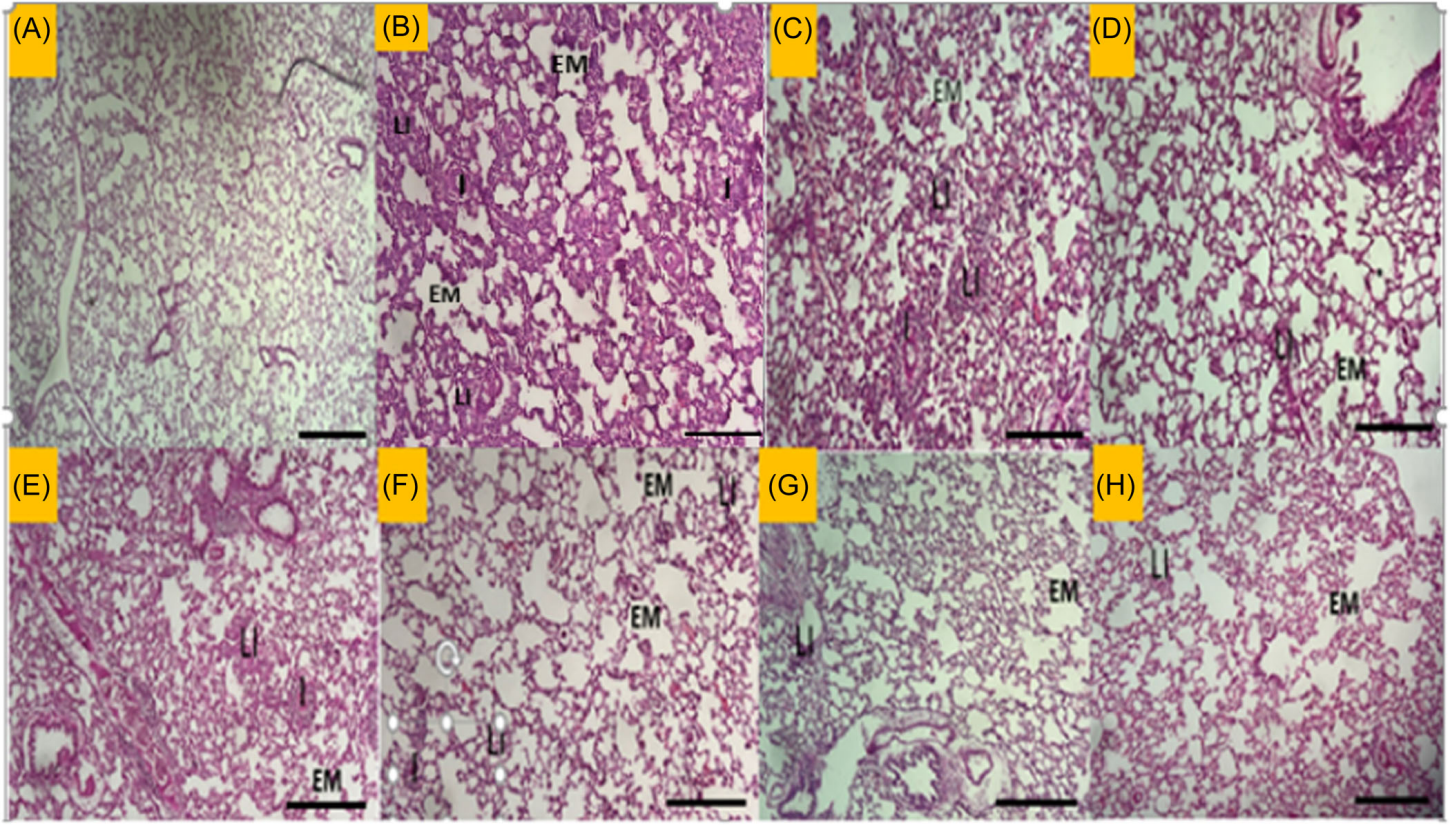
Hematoxylin and eosin (H&E) staining shows pathological changes in lung tissues in rats after PQ inhalation (Scale bar = 100 μm (magnification, ×100). (A), control group. (B), PQ‐treated group. (C), Cl‐L group. (D), Cl‐H group. (E), Cu group. (F), Cl‐L+Pio group. (G), Pio group. (H), Dex group. EM, Emphysema; I, Inflammation; LI, Lymphocyte infiltration.

PQ poisoned group showed a significant increase in lung pathology, with significant changes in interstitial inflammation, emphysema, and lymphocyte infiltration (*p* < .01 to *p* < .001). In the Cu group, the emphysema score was markedly lower than in the PQ group (*p* < .05). In all treated groups, lung interstitial inflammation and lymphocyte infiltration were markedly improved except for Cl‐L and Pio (*p* < .05 to *p* < .001), (Figure 7). The images of histopathological findings of each studied group are shown in Figure 8.

## 4 DISCUSSION

In this work, the detrimental effects of PQ inhalation on total and differential WBC, oxidative stress markers in the BALF, cytokine levels in the lung tissues, lung histologic lesions score and airway sensitivity and responsiveness to methacholine and the therapeutic effects of Cl extract, Cu, Pio, and Cl +Pio in comparison with dexa as a standard treatment, on the mentioned parameters were assessed in rats.

Increase MDA level and decreased activities of anti‐oxidant enzymes (SOD and CAT) and thiol content in the BALF of the PQ group, indicated the induction of oxidative damage to the lung tissue, which was consistent with recent reports.^36^, ^37^ Reactive oxygen species (ROS), as one of the main regulators of cellular signals, play a special role in processes such as growth, development, inflammation, and apoptosis of cells.^38^ In oxidative stress conditions such as PQ poisoning, the overproduction of ROS can change the structure and function of macromolecules.^39^, ^40^ The exact mechanisms of PQ‐induced pulmonary toxicity have not been fully understood, but it is assumed that PQ increases the production of ROS, which leads to cellular death and lung tissue damage.^41^, ^42^ Therefore, antioxidants are thought to improve PQ‐induced lung injury by inhibiting ROS production.

Treatment with Cl, Cu, Pio, Cl‐L + Pio, and Dex decreased MDA level but increased total thiol groups, CAT, and SOD activities in the BALF. Previous experimental studies have indicated the antioxidant effects of Cl,^43^, ^44^, ^45^, ^46^ Cu,^47^, ^48^ and Pio^49^, ^50^, ^51^ in various pathological condition. According to the results of our study, inhalation of PQ increased the level of IL‐10 and TNF‐α in the lung tissue. Also, BALF cellular analysis showed increased total and all differential WBC counts in PQ‐poisoned rats indicating PQ‐induced inflammatory response in the lung tissue which was in line with our previous findings^32^, ^37^, ^52^, ^53^ and other published studies.^54^, ^55^ As important components and pivotal players in the immune system, leukocytes are involved in processes related to inflammation and tissue repair. During lung damage, leukocytes transmigrate across the endothelial and epithelial barriers into the alveolar space.^56^ Under normal circumstances, the dynamic balance of pro‐ and anti‐inflammatory signals maintains an equilibrium condition that may change under the influence of pathological agents. In the present study, we measured the level of lung tissue TNF‐α as a potent inflammatory cytokine. In many pulmonary diseases, TNF‐α generation leads to the development of inflammatory responses.^57^ In line with the evidence from previous studies,^58^, ^59^, ^60^ we hypothesize that TNF‐α plays an essential role in leukocyte trafficking to the injured lungs. As an immunosuppressive cytokine, IL‐10 plays an essential role in restriction of inflammatory process in lung injury^61^ and exogenous supply of IL‐10 improved lung injury induced by LPS ^62^ and hyperoxia.^63^

Therefore, in our study, we consider the increased amount of IL‐10 in the lung tissue as an endogenous response following PQ inhalation, which can play a positive role in the lung recovery process. However, treatment with Cl, Cu, Pio, Cl‐L + Pio, and Dex reduced total and differential WBC as well as TNF‐α and IL‐10 levels. Immunomodulatory effects of Cl and Cu on lung disorders such as asthma,^64^ lung ischemia‐reperfusion,^65^ COPD‐like airway inflammation^66^ have previously been shown, which support the results of our study. In line with our previous finding in PQ‐poisoned rats,^32^, ^67^ Pio administration suppressed lung inflammation in rat model sepsis ^68^, ^69^

Based on our findings, we can consider a direct interaction between the occurrence of oxidative stress and leukocyte infiltration, which may confirm the findings of previous studies.^70^ In LPS—challenged mouse, ROS formation led to the production of chemokines in the pulmonary epithelial cell which trigger killer B cell recruitment in alveolar space.^71^ Also, based on the interdependent nature of oxidative stress and inflammation, inflammatory response following PQ toxicity can induce oxidative stress in lung tissue. As a part of this mechanism, nicotinamide adenine dinucleotide phosphate Oxidases (NOXs) present in the membrane of leukocytes in response to bacteria or foreign elements such as PQ can cause the production of oxidants in the leukocytes to protect the lung.^72^, ^73^

The results of this study also showed tracheal hyperresponsiveness in the PQ poisoned group indicated by a leftward shift in the methacholine concentration‐response curve and decreased EC_50_ value which confirm our previous evidence.^53^, ^74^ Furthermore, a clinical study has reported the occurrence of asthma attacks in patients who survived PQ poisoning.^75^ Increased TR was associated with increased oxidative stress and inflammatory response. Oxidative stress can cause functional changes such as increased contractility and induction of inflammatory mediator production in airway smooth muscle cells.^76^ For example, in guinea pigs, oxidative stress markers (H2O2 and 8‐iso‐PGF2α), trigger bronchial smooth muscle contraction in a concentration‐dependent manner.^77^, ^78^ Also, ROS facilitates the contractile effect of inflammatory agents such as TNF‐α on the airway smooth muscle.^79^

In this work, TR decreased in the treatment groups indicated by increased EC_50_ levels. The relaxant effect of Cl^80^ and Cu^81^ on TSM was reported in our previous studies. In addition, previous works showed the effects of Cl and Cu on an animal model of asthma.^33^, ^82^, ^83^ Xin Zeng et al. also reported that Cu suppresses the proliferation of airway smooth muscle in vitro and in vivo.^84^ Intranasal Cu administration suppresses airway remodeling in a murine model of chronic asthma.^85^ Furthermore, in diet‐induced obese rats, Pio decreased airway hyperactivity by inhibiting M2 receptor dysfunction.^86^ These findings supported the results of this study regarding the effect of Cl, Cu, and Pio on TR in PQ‐exposed rats. Also, histopathological change of PQ—poisoned animal showed emphysema, inflammation, lymphocyte infiltration in lung tissue which confirms structural damage and inflammatory response following pulmonary toxicity. Typically, emphysema is characterized by inflammatory processes which illustrates evidence of the activation of innate and adaptive immunity.^87^ It seems that the therapeutic agents in our study have resolved the mentioned pathological process with their anti‐inflammatory and antioxidant properties.

In the present study, the improving effects of the high dose of Cl on the studied variables were greater than its low dose which may suggest dose‐dependency effects of the plant. Also, to evaluate the effects of Cu compared to Cl extract, the effects of Cu were examined which showed comparable or even higher effects on oxidative stress, pathological change, and TR but lower effect on the cellular indices of BALF than the high dose of Cl extract. These results suggest that the effects observed for Cl are mainly due to its constituent Cu.

The Cl‐L +Pio group showed higher effects compared to Pio or extract alone. Therefore, the findings of this study show that adding Cl to Pio can improve the effects of PPARγ agonist drugs, which may suggest the stimulatory effect of Cl on PPARγ. However, to confirm this suggestion, the effect of Cl should be examined in combination with a PPARγ antagonist drug. Other limitations of this study are morphological changes (occurring in the tissues in different treatment groups i.e. apoptosis, neuroinflammatory markers) which should be examined in further studies.

## 5 CONCLUSION

The results of this research showed the pulmonoprotective effects of Cl and Cu in an experimental model of PQ poisoning, which were comparable to the therapeutic effect of Dex. The results also suggest that the effects of Cl are mainly due to its constituent Cu. The influence of Cl on the PPARγ receptor is also suggested. Since there is no specific antidote to neutralize the harmful effects of PQ, our study can provide evidence in favor of the therapeutic effects of the natural compound in improving inflammation, which is comparable to conventional treatments. However, it is necessary to design and implement future research based on the promotion of anti‐inflammatory properties of these agents in further experimental models or clinical studies.

## AUTHOR CONTRIBUTIONS

“All authors contributed to the study conception and design. Mohammad Hossein Eshaghi Ghalibaf, Mohammad Ehsan Taghavi zadeh Yazdi, Mona Mansourian, and Nema Mohammadian Roshan performed material preparation, data collection, and analysis, and Mohammad Hossein Eshaghi Ghalibaf wrote Mohammad Hossein Boskabady the first draft of the manuscript and all authors commented on previous versions of the manuscript. All authors read and approved the final manuscript.”

## CONFLICT OF INTEREST STATEMENT

The authors declare no conflict of interest.

## ETHICS STATEMENT

The Ethics Committee of Mashhad University of Medical Sciences (970790) approved the experiment protocol.

## Supporting information

Checklist_S1.

## Data Availability

All data generated or analyzed during this study are included in this published article (and its Supporting Information files).
